# *Mycobacterium leprae* Infection in a Wild Nine-Banded Armadillo, Nuevo León, Mexico

**DOI:** 10.3201/eid2803.211295

**Published:** 2022-03

**Authors:** Lucio Vera-Cabrera, Cesar J. Ramos-Cavazos, Nathan A. Youssef, Camron M. Pearce, Carmen A. Molina-Torres, Ramiro Avalos-Ramirez, Sebastien Gagneux, Jorge Ocampo-Candiani, Mercedes Gonzalez-Juarrero, Jorge A. Mayorga-Rodriguez, Leonardo Mayorga-Garibaldi, John S. Spencer, Mary Jackson, Charlotte Avanzi

**Affiliations:** Servicio de Dermatología, Hospital Universitario “José E. González,” Universidad Autónoma de Nuevo León, Monterrey, Mexico (L. Vera-Cabrera, C.J. Ramos-Cavazos, C.A. Molina-Torres, R. Avalos-Ramirez, J. Ocampo-Candiani);; Mycobacteria Research Laboratories, Colorado State University, Fort Collins, Colorado, USA (N.A. Youssef, J.S. Spencer, C.M. Pearce, M. Gonzalez-Juarrero, M. Jackson, C. Avanzi);; Swiss Tropical and Public Health Institute, Basel, Switzerland (S. Gagneux, C. Avanzi);; University of Basel, Basel (S. Gagneux, C. Avanzi);; Instituto Dermatológico de Jalisco "Dr. José Barba Rubio," Guadalajara, Mexico (J.A. Mayorga-Rodriguez, L. Mayorga-Garibaldi)

**Keywords:** leprosy, Mycobacterium leprae, nine-banded armadillo, Dasypus novemcinctus, whole-genome sequencing, Nuevo León, Mexico, tuberculosis and other mycobacteria, zoonoses, Hansen disease

## Abstract

Nine-banded armadillos (*Dasypus novemcinctus*) are naturally infected with *Mycobacterium leprae* and are implicated in the zoonotic transmission of leprosy in the United States. In Mexico, the existence of such a reservoir remains to be characterized. We describe a wild armadillo infected by *M. leprae* in the state of Nuevo León, Mexico.

Nine-banded armadillos (*Dasypus novemcinctus*) can be naturally infected with *Mycobacterium leprae* and have been implicated in the zoonotic transmission of leprosy in the US states of Texas, Louisiana, Alabama, Georgia, and Florida (*1*,*2*). Despite Mexico falling within the armadillos’ natural geographic habitat and the report of 182 new human leprosy cases in Mexico in 2019 (*3*), only 1 report of an armadillo infected with acid-fast bacilli has occurred since 1984, and the bacterial species in that case was never fully characterized (*4*).

In 2019, a nine-banded armadillo with ataxia, dyspnea, and adynamia was captured along the Pilon River in Montemorelos in the state of Nuevo León, Mexico. The animal was euthanized, and necropsy revealed granulomatous lesions in diverse organs and tissues (Appendix Figure 1). Histopathologic examination identified acid-fast bacilli in the liver, lung, heart, striated muscle, and ear; the bacilli were especially abundant in the spleen (Figure; Appendix Figure 2). We confirmed the presence of *M. leprae* in tissue by PCR testing of DNA extracted from the ear, liver, and lung by using the specific repetitive element RLEP (*5*) (Appendix). We used bacterial DNA extracted from the liver of the infected armadillo (strain A1), harboring the highest bacilli number by microscopy, for library preparation, followed by targeted enrichment using hybridization capture and whole-genome sequencing using NextSeq 500 (Illumina, https://www.illumina.com) (Appendix). The mean read coverage of 87× was sufficient for further comparative analysis at the single nucleotide level with other *M. leprae* isolates (Appendix Table 1). The armadillo-derived A1 strain belongs to genotype 3I-2, similar to other *M. leprae* isolates from the United States, Venezuela, Brazil, and Mexico (*1*).

**Figure:**
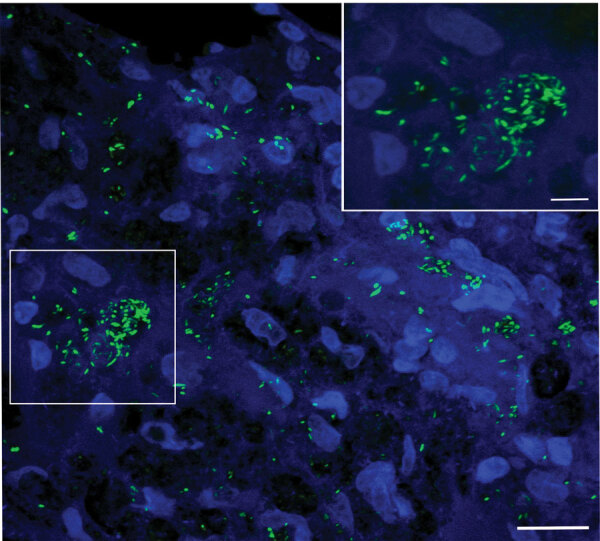
Identification and characterization of leprosy and *Mycobacterium leprae* acid-fast bacilli in the tissue in the wild nine-banded armadillo (*Dasypus novemcinctus*), Nuevo León, Mexico. SYBR gold staining shows a high density of bacilli in the spleen tissue organized in globi (boxed area at left and inset at right). Image is a merger of 16 images, 0.33 µm apart, in a z-stack taken with a 100× objective lens. Scale bars represent 20 µm (main image) and 5 µm (inset).

Phylogenetically, A1 branches between the US human (NHDP-98) and animal–human (I30, NHDP-63, NHDP-55) *M. leprae* strains and closely clusters with EGG (*6*), a strain isolated in 2014 from a 70 year-old man with leprosy living in Nuevo León, Mexico (Appendix Figure 4, 5). Strains A1 and EGG share 9 polymorphisms when compared with the whole-genome sequences from 295 other *M. leprae* isolates and differed from each other by only 5 single-nucleotide polymorphisms (SNPs) (Appendix Figure 6).

We submitted DNA from *M. leprae* isolates recovered from the biopsies of additional leprosy patients from the states of Nuevo León (n = 9) and Jalisco (n = 2), Mexico, to partial whole-genome sequencing (n = 4) and PCR genotyping (n = 7) (Appendix Table 2, Figure 5). We deciphered their clustering from previously described positions specific to genotypes 3I-1 and 3I-2 (*1*) as well as new informative SNPs specific to EGG and A1 (Appendix Table 2, Figure 6). Partial genome reconstruction for all 11 isolates revealed that 4 of them belong to genotype 3I-1, whereas 7 belong to genotype 3I-2. Within genotype 3I-1, isolates F2, F6, and F11 belong to a similar cluster, named 3I-1-c2 (Appendix Figures 4, 5). Within genotype 3I-2, 4 isolates (F1, F8, F14, and F23) belong to the same cluster, named 3I-2-c3, which also encompasses A1 and EGG. Of these isolates, only F1 shared an additional common SNP with A1 (Appendix Figures 4, 6). All patients infected with an *M. leprae* isolate from cluster 3I-2-c3 live in close vicinity (radius of ≈100 km) to the city of Montemorelos, where the infected armadillo was captured (Appendix Figure 5).

We describe the identification and genetic characterization of *Mycobacterium leprae* in a wild nine-banded armadillo in Mexico. In addition, we show that *M. leprae* strains belonging to different clusters are circulating in patients in Mexico. The state of Nuevo León, Mexico, shares a border with the US state of Texas, where a high density of leprosy-infected nine-banded armadillos have been reported (*4*,*7*). Nine-banded armadillos expanded their range into the United States in the mid-1800s from Mexico (*8*).

The *M. leprae* armadillo isolate from Mexico we describe belongs to the same genotype as patients and armadillo isolates from the United States but clusters separately. Isolate A1 further clusters with human isolates exclusively identified in Mexico thus far, with which it displays similar low genetic variation as observed between animal and human isolates in the United States (*1*). Therefore, our results raise concerns that wild-banded armadillos may, similarly to the situation in the United States, serve as reservoirs for the leprosy bacillus in the state of Nuevo León and call for additional surveillance across Mexico to assess the spread of the disease in the animal population and evaluate zoonosis risks associated with human contact with armadillos.

The existence of an animal reservoir hosting the leprosy bacillus in Mexico threatens the goal of leprosy elimination. In light of our results, we propose that interventions based on a One Health approach may be more efficient in achieving eradication of the disease.

Appendix Additional information about *Mycobacterium leprae* infection in a wild nine-banded armadillo, Nuevo León, Mexico.
