# Supplementary material for: Mycobacterium leprae Infection in a Wild Nine-Banded Armadillo, Nuevo León, Mexico
Source: Emerg Infect Dis. 2022 Mar;28(3):747–9. doi: 10.3201/eid2803.211295 (PMC8888246; doi:10.3201/eid2803.211295)
Supplement: Appendix — Additional information about Mycobacterium leprae infection in a wild nine-banded armadillo, Nuevo León, Mexico. [file 21-1295-Techapp-s1.pdf]

Article DOI: https://doi.org/10.3201/eid2803.211295

Mycobacterium leprae Infection in a Wild Nine-Banded Armadillo, Nuevo León, Mexico

Appendix

Methods

Leprosy Characterization in the Wild Banded Armadillo

Gold standard leprosy diagnosis in human relies on the identification of clinical signs of the disease and the presence of bacilli in tissues. We applied the same approach in animals by examining lesions in internal organs (Appendix Figure 1) since clinical signs are mild in wild banded armadillos (*1*), next identifying acid-fast bacilli in different tissues (Appendix Figure 2) and finally confirming *M. leprae* by molecular methods (Appendix Table 1).

Technically, assessing a zoonosis risk for leprosy relies on the genetic characterization of the strain in both the human and animal hosts. The reference work in the leprosy field is the study conducted by Truman and colleagues on wild armadillos in the United States where human and animal strains were submitted to whole-genome sequencing (WGS) before identification of specific polymorphisms (*2*). These newly identified polymorphisms were then used to screen a bigger sample set. In parallel, the direction of transmission was also deciphered from comparative genomics of *M. leprae* strains from other countries including ancient strains from Europe (*2*). We applied here a similar approach by sequencing the whole genome of the armadillo strain A1 and compared it to human *M. leprae* strains from Mexico (AGM, EGG, F6, F11 and F14) to decipher specific markers that were then used to screen more human samples from Mexico (P1, F30, F33, F1, F8, F23).

Overall, our data identified three clusters of *M. leprae* strains in Nuevo León, with half of the human strains belonging to the same cluster as the armadillo A1 strain.

Collection of Armadillo Sample

Our group has been working on genotyping samples from patients with leprosy, soil samples from armadillo burrows and samples from live animals in the state of Nuevo León, Mexico in the frame of a PhD project (*3*). During the investigations in 2019, a nine-banded armadillo presenting ataxia, dyspnea, and adynamia was captured in El Ejido San Francisco, in the vicinity of the Pilon River in Montemorelos, a municipality of Nuevo León (NL), Mexico. The animal was euthanized, and autopsy revealed granulomatous lesions in diverse organs and tissues (Appendix Figure 1). At the time of the necropsy, samples of internal (brain, liver, lung, heart, striated muscle, lymph nodes, spleen) and external organs (ear) were aseptically removed and prepared for histological examination as well as preserved in 70% ethanol for further molecular investigation. A piece of ear was collected because the animal had lost the tips of its ears (Appendix Figure 1) and while this could be the results of an injury, the team was wondering if this could be linked with leprosy lesions. The microscopy result shows the presence of acid-fast bacilli in the ear of the animal suggesting that the ear could be used as a sample for screening.

Staining of *M. leprae* in Armadillo Tissues to Detect Acid-Fast Bacilli

Tissue samples of all organs were fixed in 4% paraformaldehyde in phosphate buffered saline (PBS) and sectioned to 8–10 µm thickness. Subsequent tissues were mounted on glass slides and stained using Fite Faraco staining as previously described (*4*).

Additional tissue from spleen, liver and lung were embedded in paraffin and sent to Colorado State University where SYBR Gold fluorescent staining was performed as previously described (*5*). Images were taken on an LSM 510 Confocal microscope with a 405 nm laser for DAPI and a 488 argon laser for SYBR gold.

DNA Extraction of Armadillo and Human Samples

DNA isolation from fresh tissue samples (AGM, all samples with names starting with an F, and armadillo tissues: liver, spleen, lung, ear, heart, muscle) was carried out as previously described by Van Embden and colleagues (*6*). DNA extraction from internal organs was prioritized for genomic analysis given the high number of acid-fast bacilli found in liver of the infected animal. For paraffin-embedded samples (P1), DNA extraction was carried out on 12 µm cuts using a new blade for each sample to avoid contamination. Samples were first

deparaffinized using xylene and ethanol followed by bacterial lysis with proteinase K at 56°C for 3h and DNA purification the Qiagen DNeasy kit (Qiagen, Germany) as per the manufacturer's recommendations. The quality and quantity of DNA was then assessed using the Eppendorf biophotometer.

Detection of RLEP Sequences by PCR and Quantitative PCR

Detection by PCR (CNR, Mexico)

Characterization of the leprosy agent in human skin biopsies and animal samples was performed using the specific primers targeting the 488-bp region of the core RLEP (*M. leprae*) and the 244-bp region of *hemN* (*M. lepromatosis*) as previously described (*7*). The results are shown in the gel picture Appendix Figure 3 for the liver, lung and ear sample from the wild armadillo.

Detection by qPCR (CSU, USA)

Quantitative PCR was performed on sample A1 extracted from the spleen of the armadillo, as well as on all DNA extracted from human skin biopsies using the specific RLEP sequence as described previously (*8*). qPCR data are shown in Appendix Figure 6.

Whole-genome Sequencing, Data Analysis, and Comparative Genomics

DNA libraries from A1, F6, F11, F14 and AGM were prepared using the Kapa Hyper Prep kit (Roche, Switzerland) as per the manufacturer's recommendation using Kapa Dual Indexed Adaptor (Roche, Switzerland) followed by in-solution capture enrichment with 80-nt RNA baits with 2x tiling density for 48h at 65°C as previously described (*8*). Post-capture amplification was performed with seven cycles. Enriched libraries were purified using a 1X ratio of KAPA Pure beads followed by quantification with the KAPA library quantification kit and quality control of the fragment with the Agilent 2200 TapeStation. Libraries were then normalized and pooled across sequencing lanes on an Illumina NextSeq 500 on a high output kit v2; 75 cycles (Illumina, CA, USA). Raw reads were processed as described elsewhere and sequencing parameters for each genome are shown in Appendix Table 1 (*8*). Phylogenetic analyses were performed using a concatenated SNP alignment. Maximum Parsimony (MP) trees were constructed in MEGAX (*9*) with the 295 *M. leprae* genomes available on public databases (*10*) using 500 bootstrap replicates and *M. lepromatosis* (*7*) as outgroup. Sites with missing data

were partially deleted (80% coverage cutoff), resulting in 4,567 variable sites used for the tree calculation.

Genotyping of *M. leprae* Strains and Amplification of Specific Loci

The comparative genomics of all sequenced strains from Mexico (A1, F6, F11, F14, AGM, EGG) with the 295 publicly available *M. leprae* genomes revealed that Mexican strains belong to three different clusters in the genotype 3I that we arbitrary named 3I-1-c1; 3I-1-c2 and 3I-2-c3. SNP of the sequenced *M. leprae* genomes from each cluster were compared to the 295 publicly available *M. leprae* genomes and specific loci for each cluster were identified (Appendix Figure 4). To identify genotype-specific SNPs in samples where WGS was not possible (F1, F2, F6, F8, F11, F14, F23), due to high qPCR Cycle threshold (Ct) (>25), we amplified a handful of genomic positions according to the genotyping scheme shown in Appendix Figure 6. To do so, primers were designed using the Primer3 web tool and are described in Appendix Table 2. For each sample, 5 µl of the starting materials, negative control (water) or positive control (*M. leprae* DNA strain Thai-53, NR19352) was used in 50 µl reactions using the Quick-Load Tas 2X Master Mix, and quality was assessed on 1X agarose gel. Amplification started with a 3 min initial denaturation step at 94°C, followed by 40 cycles of 30 s denaturation at 94°C, 30 s annealing at 58°C (all PCR primers' optimized temperatures are in Appendix Table 2), and extension at 72°C for 30 s; final extension was at 72°C for 5 min. Amplicons were purified using ExoSAP-IT PCR Product Cleanup reagent and sequencing was done by Genewiz.

Genotyping of F1

Our data show that the strain F1 harbors the genotype 3I-2 and belongs to the cluster 3I-2-c3 (SNP-7614 and SNP-3260610). Next, to differentiate strains within cluster 3I-2-c3, we identified SNPs specific to either A1 or EGG. *M. leprae* is a clonal organism with limited expected genetic variation between strains from the same geographic area (*11*,*12*), and the SNP-1147347 is specific to A1 (not found in any of the 295 other genomes analyzed). Therefore, despite having a partial genome reconstruction for F1, the fact that the strain harbors the SNP-7614, SNP-3260610 and SNP-1147347 and the absence of SNP-1147262 and SNP-1206419 is indicative of the strain belonging to the cluster 3I-2-c3 that will branch more closely to A1 than to F14 and EGG.

Genotyping of F8 and F23

Both strains harbor the genotype 3I-2 and also belong to the cluster 3I-2-c3 (SNP-7614 and SNP-3260610). However, they do not share any of the specific SNPs identified in A1 or EGG so we cannot further decipher the branching of these two strains inside the cluster 3I-2-c3.

Genotyping of F6 and F11

The strains F6 and F11 belong to the genotype 3I-1 (SNP-7614, absence of SNP-1527053) and form a cluster (named 3I-1-c2) between VB-21 (Venezuela) and Br2016–46 (Brazil). We identified one SNP (SNP-732918) which is only shared by F6 and F11 and not by any of the other 295 genomes used for the comparative genomic.

Data Availability

All raw read files have been deposited in the trace archive of the National Center for Biotechnology Information (NCBI) Sequence Read Archive (SRA) accession number SRR14638043-SRR14638047; under the BioProject accession PRJNA732526 (https://dataview.ncbi.nlm.nih.gov/object/PRJNA732526?reviewer = 3scakbpm47kpvolo6n0konjg4u).

Ethics Statement

Ethics approval from the ethical committee from the Hospital Universitario "Dr. José Eleuterio Gonzalez" (number DE19–00010) to collect all clinical samples and perform molecular investigations of the *M. leprae* strains. Written informed consent was obtained from all the patients for biopsy procedure and data sharing.

References

1. Truman R. Leprosy in wild armadillos. Lepr Rev. 2005;76:198–208. PubMed https://doi.org/10.47276/lr.76.3.198

2. Truman RW, Singh P, Sharma R, Busso P, Rougemont J, Paniz-Mondolfi A, et al. Probable zoonotic leprosy in the southern United States. N Engl J Med. 2011;364:1626–33. PubMed https://doi.org/10.1056/NEJMoa1010536

3. Ramos Cavazos CJ. Epidemiología molecular de mycobacterium leprae y mycobacterium lepromatosis en el estado de Nuevo León. 2020 [cited 2021 May 21]. http://eprints.uanl.mx/20518

4. Vera-Cabrera L, Escalante-Fuentes WG, Gomez-Flores M, Ocampo-Candiani J, Busso P, Singh P, et al. Case of diffuse lepromatous leprosy associated with "*Mycobacterium lepromatosis*". J Clin Microbiol. 2011;49:4366–8. PubMed https://doi.org/10.1128/JCM.05634-11

5. da Silva MB, Portela JM, Li W, Jackson M, Gonzalez-Juarrero M, Hidalgo AS, et al. Evidence of zoonotic leprosy in Pará, Brazilian Amazon, and risks associated with human contact or consumption of armadillos. PLoS Negl Trop Dis. 2018;12:e0006532. PubMed https://doi.org/10.1371/journal.pntd.0006532

6. van Embden JD, Cave MD, Crawford JT, Dale JW, Eisenach KD, Gicquel B, et al. Strain identification of *Mycobacterium tuberculosis* by DNA fingerprinting: recommendations for a standardized methodology. J Clin Microbiol. 1993;31:406–9. PubMed https://doi.org/10.1128/jcm.31.2.406-409.1993

7. Singh P, Benjak A, Schuenemann VJ, Herbig A, Avanzi C, Busso P, et al. Insight into the evolution and origin of leprosy bacilli from the genome sequence of *Mycobacterium lepromatosis.* Proc Natl Acad Sci U S A. 2015;112:4459–64. PubMed https://doi.org/10.1073/pnas.1421504112

8. Avanzi C, Lécorché E, Rakotomalala FA, Benjak A, Rapelanoro Rabenja F, Ramarozatovo LS, et al. Population genomics of *Mycobacterium leprae* reveals a new genotype in Madagascar and Comoros. Front Microbiol. 2020;11:711. PubMed https://doi.org/10.3389/fmicb.2020.00711

9. Kumar S, Stecher G, Li M, Knyaz C, Tamura K. MEGA X: Molecular Evolutionary Genetics Analysis across Computing Platforms. Mol Biol Evol. 2018;35:1547–9. PubMed https://doi.org/10.1093/molbev/msy096

10. Tió-Coma M, Avanzi C, Verhard EM, Pierneef L, van Hooij A, Benjak A, et al. Genomic characterization of *Mycobacterium leprae* to explore transmission patterns identifies new subtype in Bangladesh. Front Microbiol. 2020;11:1220. PubMed https://doi.org/10.3389/fmicb.2020.01220

11. Monot M, Honoré N, Garnier T, Zidane N, Sherafi D, Paniz-Mondolfi A, et al. Comparative genomic and phylogeographic analysis of *Mycobacterium leprae.* Nat Genet. 2009;41:1282–9. PubMed https://doi.org/10.1038/ng.477

12. Benjak A, Avanzi C, Singh P, Loiseau C, Girma S, Busso P, et al. Phylogenomics and antimicrobial resistance of the leprosy bacillus *Mycobacterium leprae.* Nat Commun. 2018;9:352. PubMed https://doi.org/10.1038/s41467-017-02576-z

13. Yuan S, Chan HCS, Filipek S, Vogel H. PyMOL and Inkscape bridge the data and the data visualization. Structure. 2016;24:2041–2. PubMed https://doi.org/10.1016/j.str.2016.11.012

Appendix Table 1. Sequencing data for all five newly sequenced genomes from nine-banded armadillo (A1) and humans (F6, F11, F14 and AGM)

Sample name	Number of reads mapping to the reference strain (TN)	Mean Coverage	Mean Standard Coverage	SRA accession
A1	21,290,001	86.9	16.8	SRR14638047
F6	42,457,775	5.25	6.1	SRR14638046
F11	39,047,908	2.5	3.5	SRR14638045
F14	40,575,889	6.6	5.4	SRR14638044
AGM	59,336,213	85.5	24.4	SRR14638043

Appendix Table 2. List of primers used in this study – Opt T°C: optimal annealing temperature for the corresponding primers set

Primer name	Target gene	Purpose	Amplicon size (bp)	Primer sequence (5′-3′)	Nucleic acid modification	Opt T°C
131F	*ml0978*	Specific to A1 Specific to EGG	174	GCACAATCGTGAACCGACTA	A1147347G C1147262T	58°C
131R				CGACCCCGATATCGTATGAC		
132F	*ml2704*	Specific to cluster 3I-2-c3	175	TAATGATCAGCTTGCGTTGC	C3260610T	58°C
132R				CGACCGTGTGTACGACAATC		
133F	*cydD*	Specific to 3I-2	236	GCACTGATCACACTGCCATT	G1527053C	58°C
133R				GTGATCCGGAGTGTCGCTAT		
134F	*ml2681*	Specific to A1	157	TGGGACGTTTATCTGCAGTG	G3232319 G>A	58°C
134R				GGATGACGGTAGACGTTGCT		
135F	*dnaK-ml2497*	Specific to A1	241	TCTGTTACCGTGAGGGGCTA	C2974107T	58°C
135R				ACTAGTGCGGTTTGCGTCTT		
137F	*ddp*	Specific to 3I-2	261	CAATCGCAGGAGTTCACAAA	G2714879C	58°C
137R				CCTTGGCACCCTGTTACACT		
138F	*ml0600-ml0601*	Specific to cluster 3I-1-c2	273	CACCAATGCAGTTGCTGAGT	T732918G	58°C
138R				CACGCAAAGTGGCTTGATTA		
140F	*cydD*	Specific to NHDP-98	250	CAAGCGATCGCCAGACTAAC	G1526442C	58°C
140R				CCATCCCGTACGGTAAATTG		
gyrA_OMS_F	*gyrA*	Specific to 3I genotype	225	ATGGTCTCAAACCGGTACATC	C7614T	55°C
gyrA_OMS_R				TACCCGGCGAACCGAAATTG		

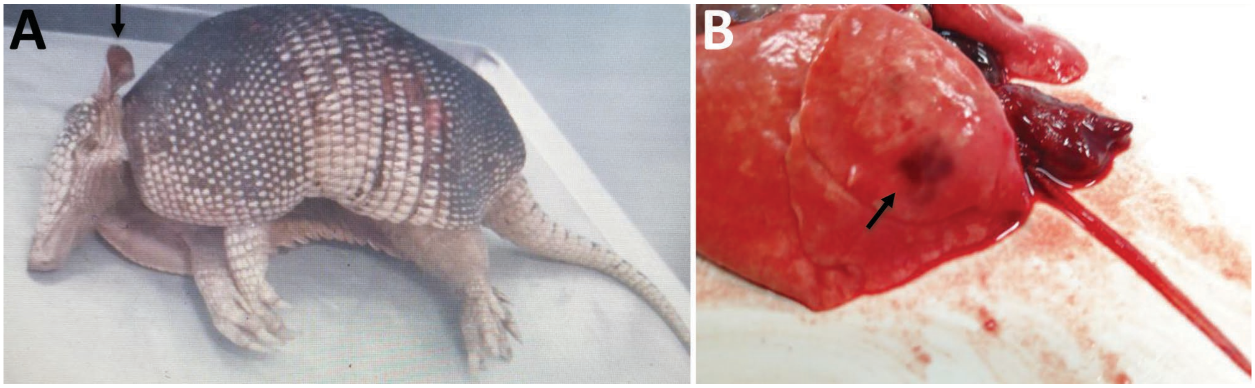

Appendix Figure 1. Macroscopic lesions of the infected nine-banded armadillo. A. Picture of the animal before autopsy - the black arrow indicates where the sampling was performed on the ear. B. Macroscopic lesions in the lungs (black arrow).

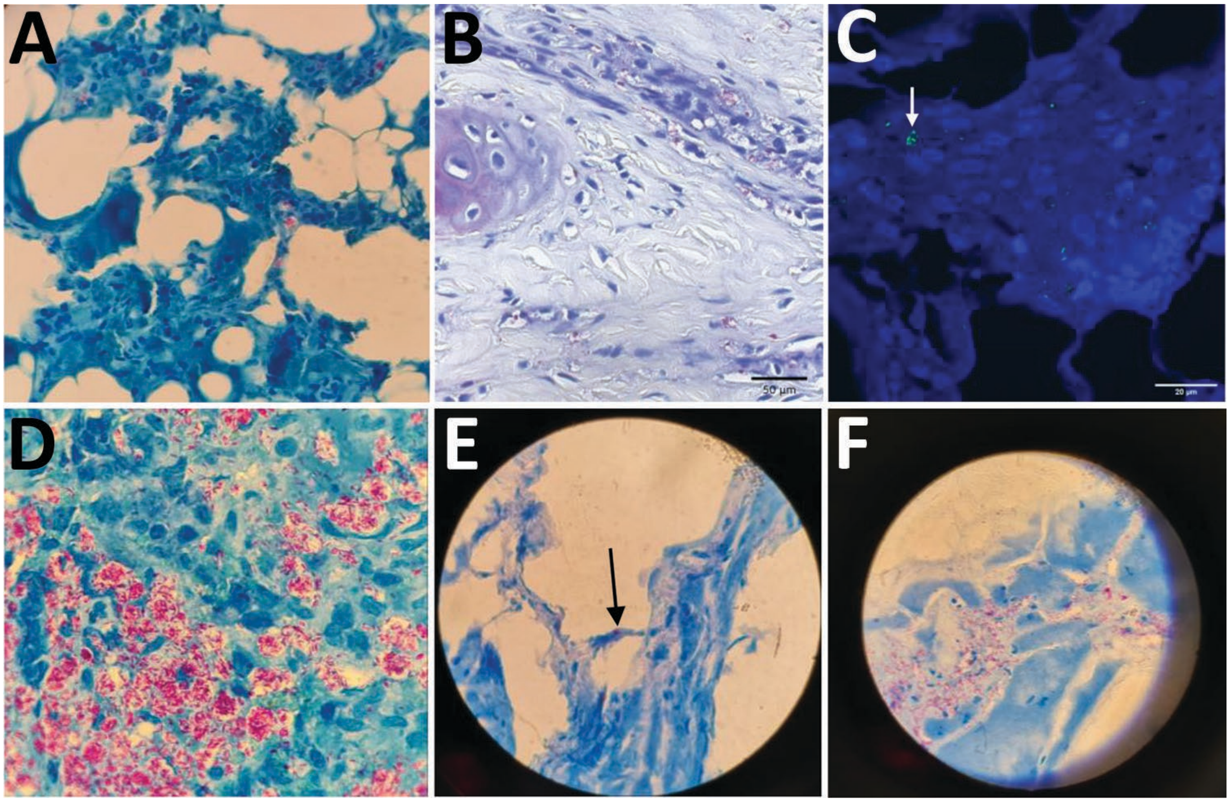

Appendix Figure 2. Fite-Faraco (A, B, D, E, F) or SYBR Gold (C) staining showing abundant acid-fast bacilli (fuchsia color in panels A., B. and D., and in green in panel C.) in A. the lung, B. the ear, C. the liver, and particularly in D., the spleen, E., heart, and F., striated muscle.

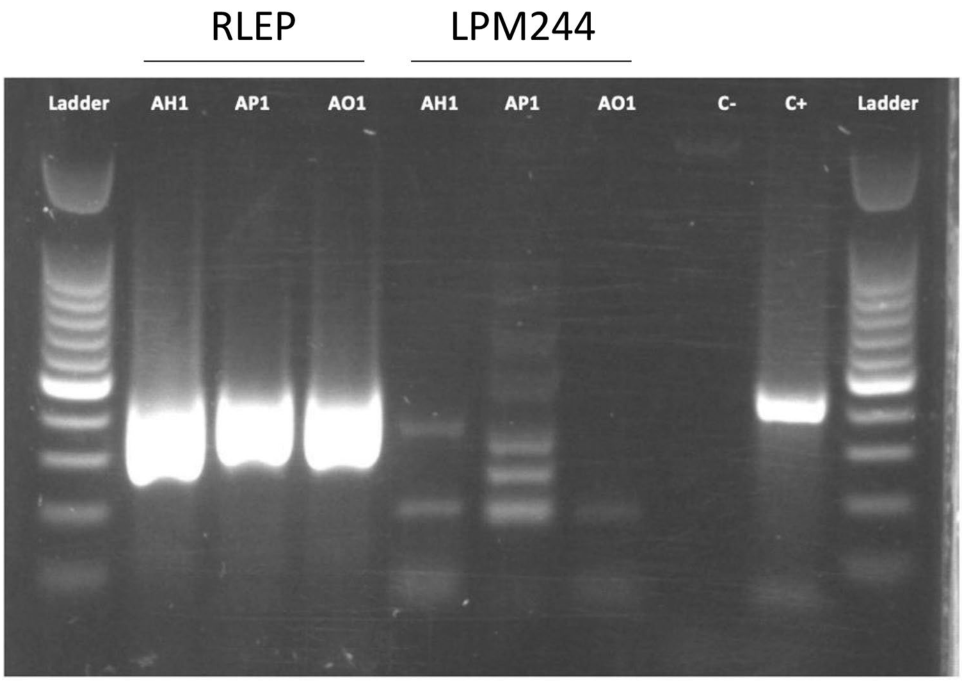

Appendix Figure 3. Results of the PCR RLEP and LPM244 performed on DNA extracted from the internal organs of the wild-banded armadillo – Two µl of undiluted DNA from the liver (AH1), lung (AP1) and ear (AO1) was used as starting materials for PCR. C-: negative control (water); C+ positive control (diluted DNA from a previously diagnosed leprosy patient) – Positive control for LPM244 is not shown in the picture and no bands are observed at 244bp.

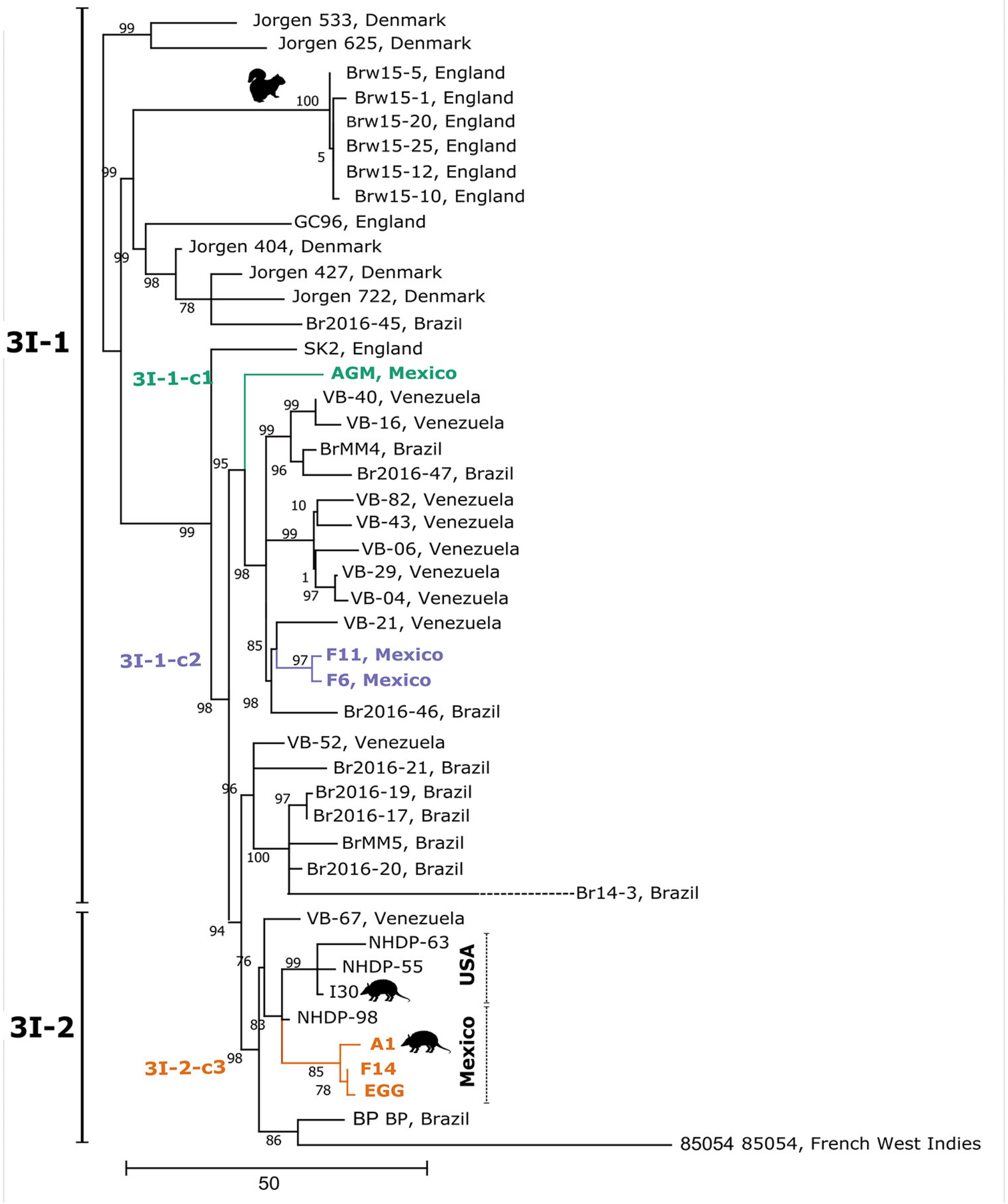

Appendix Figure 4. Comparative genomics of the *M. leprae* strain from the wild animal (A1) and the ones identified in human in Mexico (EGG, F14, AGM, F6 and F11) - The tree represents a zoom into the *M. leprae* genotypes 3I-1 and 3I-2 deciphered from a maximum parsimony tree of 300 *M. leprae* genomes. Support values were obtained by bootstrapping 500 replicates. Branch lengths are proportional to nucleotide substitutions. All genomes from Mexico are presented in bold and color-coded according to their respective cluster: 3I-1-c1 (green), 3I-1-c2 (purple) and 3I-2-c3 (orange).

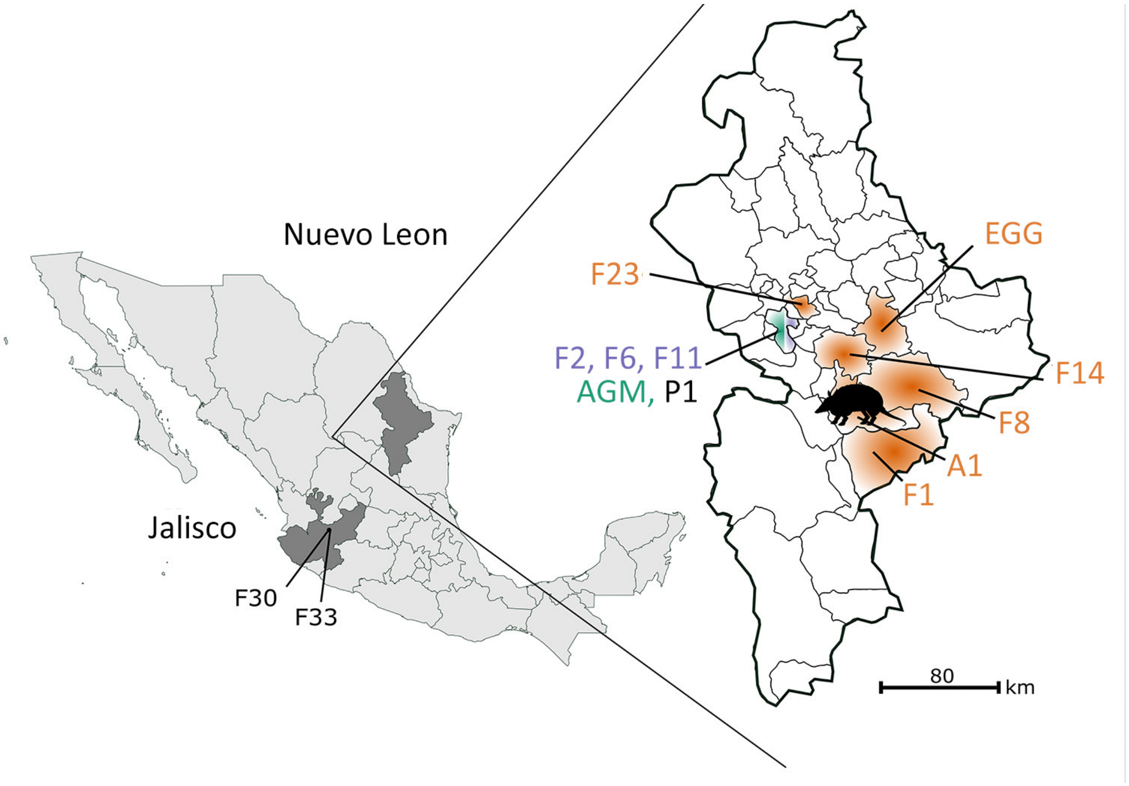

Appendix Figure 5. Additional genotyping on human *M. leprae* strains and their sampling sites in Mexico - The isolates described in this study are bold and color-coded based on genotyping results (by PCR or WGS) and their respective clusters as in B. Cluster level is unavailable for P1, F30 and F33 and data used for the map are available in Appendix Figure 6. The map was downloaded from https://www.amcharts.com/svg-maps/ under a free license; the silhouettes were downloaded from http://phylopic.org/about under a free license. The current figure was modified and colored in Inkscape (*13*).

Genome position - gene	Ct RLEP qPCR	7614 - gyrA (gyrA_OMSFR)	1527053 -cydD (133FR)	2714879 - dppD (137FR)	12046 - ml0007	14477 - ml0009-ml0010	55736 - ml0045	445671 - fadD19	817228 - metA	881107 - phoS1	2165386 - ml1790/ml1791	3009987 - ml2527	3260610 - ml2704 (132FR)	2974107 - dnaK/ml2497 (135FR)	3232319 - ml2681 (134FR)	1147347 - ml0978 (131FR)	1147262 - ml0978 (131FR)	1206419 - ml1040	732918 - ml0600/ml0601	1526442 -cydD (140FR)	Genotype	Branch	Origin of the patient	Publications associated with the strain
TN		C	G	T	C	T	G	A	C	A	G	G	C	C	G	A	C	C	T	G	1A	1	India	https://doi.org/10.1038/35059006
Br2016-47		T	G	T	C	T	G	A	C	A	G	G	C	C	G	A	C	C	T	G	3I-1	3	Brazil	https://doi.org/10.1038/s41467-017-02576-z
Br2016-17		T	G	T	C	T	G	A	C	A	G	G	C	C	G	A	C	C	T	G	3I-1	3	Brazil	https://doi.org/10.1038/s41467-017-02576-z
AGM	21.2	T	G	T	C	T	G	A	C	A	G	G	C	C	G	A	C	C	T	G	3I-1	3	Monterrey, NL, Mexico	this study
F11	24.6	T	G	T	C	T	G	A	C	A	G	G	C	C	G	A	C	C	G	G	3I-1	3	Monterrey, NL, Mexico	this study
F6	25	T	G	T	C	T	G	A	C	A	G	G	C	C	G	A	C	C	G	G	3I-1	3	Monterrey, NL, Mexico	this study
F2	29.8	T	neg	T									C						G		3I-1	3	Monterrey, NL, Mexico	this study
I30		T	C	A	C	T	G	A	C	A	G	G	C	C	G	A	C	C	T	G	3I-2	3	USA	http://doi.org/10.1056/NEJMoa1010536
NHDP-98		T	C	A	C	T	G	A	C	A	G	G	C	C	G	A	C	C	T	C	3I-2	3	Mexico	http://doi.org/10.1056/NEJMoa1010536
P1	28.1	T	C	neg									C						T		3I-2	3	Monterrey, NL, Mexico	this study
F30	26.8	T	C	A									neg	C	G	A	C			G	3I-2	3	Guadalajara, JA, Mexico	this study
F33	26.4	T	C	A									C	C	G	A	C			G	3I-2	3	Guadalajara, JA, Mexico	this study
A1	20.8	T	C	A	T	C	T	G	T	G	A	T	T	T	A	G	C	C	T	G	3I-2	3	Montemorelos, NL, Mexico	this study
F1	29.8	T	ND	neg									T	neg	G	G	C				3I-2	3	Linares, NL, Mexico	this study
EGG		T	C	A	T	C	T	G	T	G	A	T	T	C	G	A	T	T	T	G	3I-2	3	Los Ramones, NL, Mexico	https://doi.org/10.1038/s41467-017-02576-z
F14	26	T	C	A	T	C	NC	G	T	G	A	NC	T	C	G	A	T	C	T	G	3I-2	3	Cadereyta, NL, Mexico	this study
F23	26.3	T	C	neg									T	C	G	A	C				3I-2	3	Apocada, NL, Mexico	this study
F8	28.4	T	C	neg									T	C	G	A	C				3I-2	3	General Teran, NL, Mexico	this study

NC Not covered; ND Not done low DNA; Not done
3I-1 Cluster 31-1-c1; 3I-1 Cluster 31-1-c2; 3I-2 Cluster 31-2-c3
G Deciphered by WGS; SNP; neg Negative

Appendix Figure 6. Demographic and genotype of *M. leprae* isolates from human and armadillo. Previously published *M. leprae* genomes from genotypes 3I-1 (Br2016–47, Br2016–17) and 3I-2 (I30, NHDP-98, EGG) are also indicated in the figure. Ct: cycle threshold.; ND: not done; insufficient DNA quantity.
